# Differential diagnosis of non-diffuse primary thyroid lymphoma and papillary thyroid carcinoma by ultrasound combined with computed tomography

**DOI:** 10.1186/s12885-022-10035-2

**Published:** 2022-08-31

**Authors:** Chanjuan Peng, Dan Yi, Ying Zhou, Jincao Yao, Bo Chen, Chen Yang, Dong Xu

**Affiliations:** 1 Department of Ultrasound, Cancer Hospital of the University of Chinese Academy of Sciences (Zhejiang Cancer Hospital), No.1 East Banshan 7 Road, Gongshu District, Hangzhou, 310022 China; 2 Institute of Basic Medicine and Cancer (IBMC), Chinese Academy Of Sciences, Hangzhou, 310022 China; 3 Department of Ultrasound, Shaoxing People’s Hospital, Hangzhou, 312000 China; 4 Department of Surgery, Hebei Provincial Hospital of Traditional Chinese Medicine, Shijiazhuang, 050000 China; 5 Department of Radiology, Shaoxing People’s Hospital, Shaoxing, 312000 China; 6 Key Laboratory of Head & Neck Cancer Translational Research of Zhejiang Province, Hangzhou, 310022 China; 7 Zhejiang Provincial Research Center for Cancer Intelligent Diagnosis and Molecular Technology, Hangzhou, 310022 China

**Keywords:** Sonography, CT, Primary thyroid lymphoma, Papillary thyroid carcinoma

## Abstract

**Background:**

Primary thyroid lymphoma (PTL) and papillary thyroid carcinoma (PTC) are both thyroid malignancies, but their therapeutic methods and prognosis are different. This study aims to explore their sonographic and computed tomography(CT)features, and to improve the early diagnosis rate.

**Methods:**

The clinical and imaging data of 50 patients with non-diffuse PTL and 100 patients with PTC confirmed by pathology were retrospectively analysed.

**Results:**

Of the 150 patients, from the perspective of clinical data, between non-diffuse PTL and PTC patients existed significant difference in age, maximum diameter of nodule, asymmetric enlargement and Hashimoto’s thyroiditis (*P* < 0.001), but not in gender ratio, echo texture, cystic change and anteroposterior-to-transverse ratio (*P* > 0.05). With respect to sonographic feature, non-diffuse PTL patients had a higher proportion than PTC patients in markedly hypoechoic, internal linear echogenic strands, posterior echo enhancement, rich vascularity, lack of calcification and homogeneous enhancement, with statistically significant difference (*P* < 0.05), while PTC patients had a higher proportion than non-diffuse PTL patients in irregular border, circumscribed margin, capsular invasion and significant enhancement, with statistically significant difference (*P* < 0.001). With respect to CT feature, non-diffuse PTL patients were significantly different from PTC patients in the non-contrast CT value mean, venous phase CT value mean, enhanced intensity and homogeneity of nodules (*P* < 0.05). Multivariate logistic regression analysis showed that age (OR = 1.226, 95%CI:1.056 ~ 1.423, *P* = 0.007), posterior echo enhancement (OR = 51.152, 95%CI: 2.934 ~ 891.738, *P* = 0.007), lack of calcification (OR = 0.013, 95%CI: 0.000 ~ 0.400, *P* = 0.013) and homogeneous enhancement (OR = 0.020, 95%CI: 0.001 ~ 0.507, *P* = 0.018) were independent risk factors.

**Conclusions:**

Sonographic and CT features of the presence of posterior echo enhancement, lack of calcification and homogeneous enhancement were valuable to distinguishing non-diffuse PTL from PTC.

## Introduction

Primary thyroid lymphoma (PTL) is a malignant tumor originating from thyroid lymph tissue. The incidence rate is low, accounting for 5% of all thyroid malignancies and less than 3% of extranodal lymphoma [1, 2]. Thyroid lymphoma mostly occurs at 60–70 years old and the incidence rate of it is higher in the female than in the male. PTL is usually characterized as B-cell-derived Non-Hodgkin lymphoma, the most common types of which include diffuse large B cell lymphoma (DLBCL) and mucosa-associated lymphoid tissue lymphoma (MALT). This disease is closely associated with Hashimoto thyroiditis (HT) and develops in the setting of chronic thyroiditis [3–5]. The diagnosis of HT is debated and is traditionally considered histological [6, 7]. Seronegative HT is also possible, and some scoring systems were proposed [8]. Sonographic features of HT are often considered a heterogeneity hypervascularity, decreased echogenicity and presence of hypoechoic micronodules with echogenic rim [9]. Liu et al. found that combined clinical and pathological analyses with ultrasonography will help distinguish IgG4 HT from PTL when both presents with a rapidly enlarged thyroid [10]. Some scholars, such as Xia [11], have classified PTL sonography appearances into two types: diffuse and non-diffuse. Diffuse-type involves two lobes with no clear boundary, while non-diffuse focally involves one or both lobes, in which one or multiple lesion(s) nodularly or patchily present. Until now, the preoperative diagnosis rate of PTL by ultrasound is low, and the result is easily misinterpreted as thyroid cancer or HT.

Papillary thyroid carcinoma (PTC), the most common pathological type of thyroid malignancies, accounts for 80% of the total thyroid cancer, the incidence rate of which has been increasing in the late decades. With slow progression and low degree of malignancy, PTC is more common among young and middle-aged women. Previous studies showed that the incidence of PTC capsular invasion was high, which affected the risk stages of thyroid cancer and then increased the risk of recurrence [12–14]. Early diagnosis is thus particularly important.

PTL and PTC differ in the therapeutic method, although they are both thyroid malignancies. Zheng et al. have reported that clinical characteristics and sonographic features were helpful for diagnosis of PTL and diffuse sclerosing variant of papillary thyroid carcinoma (DSVPTC) before surgery [15]. The treatment for PTL is mainly chemotherapy and radiotherapy, with not so expected benefit of surgical resection [2, 16], while surgery is the preferred treatment for PTC [17, 18]. High frequency ultrasonography is the first choice of thyroid disease examination [19, 20], and studies [5, 21] have shown that CT is a supplementary diagnostic technique for thyroid diseases, due to its advantages in determining the location and range of tumors as well as its superiority to ultrasound in evaluating the infiltration range. Tomohiro et al. have reported that the CT imaging features including the maximum diameter, morphological patterns, thickening of the isthmus, invasion of surrounding tissues, regional lymphadenopathy and preserved peripheral thyroid tissue were useful in differentiating DLBCL from MALT lymphoma of the thyroid gland [22]. However, currently few reports have been published on the difference between PTL and PTC patients in sonographic and CT features. Therefore, focusing on the comparative analysis of the sonographic and CT features of nodules between non-diffuse PTL and PTC, this study aims to provide reference for early clinical diagnosis and treatment and then avoid unnecessary radical thyroidectomy.

## Material and methods

### Patient selection

**Fig. 1:**
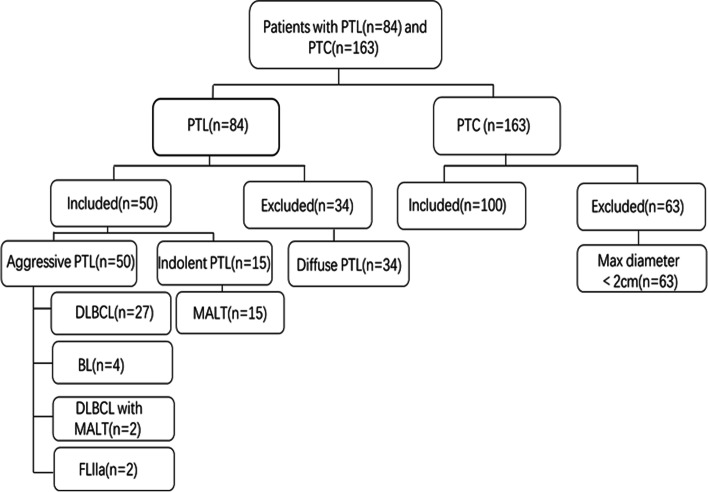
Flow chart of study participants

From a retrospective review of the pathological database of our hospital from June 2008 to September 2021, 84 patients with PTL and 163 randomly selected patients with PTC with a maximum diameter larger than 2 cm were included in our present study. Our inclusion criteria are as follows: (1) PTL or PTC that was confirmed by fine-needle aspiration cytology (FNAC) or surgical pathology; (2) non-diffuse PTL in terms of the sonographic classification, and with the maximum diameter of PTC nodules larger than 2 cm; (3) thyroid enhancement CT examination performed within two weeks before operation; (4) with detailed and complete clinical and imaging data, and no previous systemic lymphoma involving the thyroid. Patients were excluded if they had a history of systemic lymphoma and thyroid surgery, or if they had no complete clinical and imaging data. Finally, 50 patients with non-diffuse PTL and 100 patients with PTC were enrolled in this study (see Fig. 1). All their data were approved and reviewed by our hospital ethics committee (No: IRB-2020–287) in accordance with relevant guidelines and obtained through the clinical electronic medical record system. Patient records were anonymized and deidentified before analysis. Informed consent was obtained from all subjects or their legal guardian(s). The pathological results were observed and diagnosed by a physician with more than 5 years of pathological experience, and then reviewed by an expert with more than 10 years of pathological experience. All the pathological diagnoses were classified according to the 2016 WHO classification standard [23].

### Thyroid ultrasonography

Siemens S3000, Philips IU22 and GE LogiqE9 ultrasonic diagnostic instruments were used, and the probe frequency was 5-12 MHz. Thyroid sonography was performed by experienced technicians who were blind to the design of this study. Two experienced ultrasound physicians independently analyzed the images using double-blind method. Successively, they examined the size, border, margin, internal texture, internal echo, cystic change, calcification, anteroposterior-to-transverse ratio, posterior echo, vascularity and capsular invasion of thyroid nodules. Once divergence happened on ultrasonic signs, they initiated discussion and analysis and then made decision. According to the echo of lesions, the data were classified into three categories [11]: ① markedly hypoechoic: lower than the echo from anterior cervical banded muscle; ② hypoechoic: between the echo from anterior cervical banded muscle and that from normal thyroid parenchyma; ③ equal or high echo: similar to or higher than the echo from normal thyroid parenchyma. Internal linear echogenic strands are defined as line-like hyperechoic within the lesion [24]. Anteroposterior-to-transverse ratio refers to the vertical height of the nodule versus the horizontal width of the nodule [25]. Asymmetric thyroid enlargement is when the left lobe of the thyroid is larger than the right lobe or the right lobe is larger than the left lobe. Capsular invasion refers to invasion of the thyroid capsule, indicating external expansion of the thyroid. According to the classification of Adler Grades of Blood Flow [26], the images included: ① Grade-0, no blood flow signal; ② Grade-I, a small amount of blood flow and 1 or 2 dot-like or short rod blood flow signals; ③ Grade-II, moderate blood flow, 3 or 4 dot-like or 1 longer blood vessel in the lesion, and with a length and diameter approaching or exceeding the radius of the lesion; Grade-III, rich blood flow, ≥ 5 visible dot-like or 2 long blood vessels. Moreover, this study classified grades 0-I as absence/not vascularity and grades II-III as rich vascularity.

### Thyroid enhanced CT examination

Thyroid enhanced CT examination is a routine procedure in our center for the evaluation of thyroid nodules before FNAC or surgery. 64 row spiral CT machine was used for scanning, the models of which included Siemens SOMATOM Definition Flash, Siemens sensation 16 and GE MEDICAL SYSTEMS Bright Speed. The CT scan parameters adopted in this study were as follows: 120kv; 200-250 mA; matrix size, 512 × 512; pitch, 1.0; layer thickness, 3 mm or 3.8 mm. Single phase scanning was performed 45-55 s after contrast-scanning intravenous injection of non-ionic contrast agent iohexol through elbow vein. The value means of thyroid nodules in plain scanning stage and venous stage were measured by two senior radiologists. During the process, a clear section of the lesion was selected to avoid calcification and artefact, the circular region of interest (ROI) of more than 30mm^2^ in diameter was placed in five regions of lesions with homogeneous density, and the mean CT value was read [1]. According to Liu et al. [27], CT enhancement was classified into: mild enhancement, < 20HU; moderate enhancement, 20-50HU; significant enhancement, > 50HU. In this study, the enhancement < 50HU was classified as mild to moderate, and that > 50HU as significant.

### Statistical analysis

Statistical analyses were performed with SPSS version 25.0 software (IBM Corporation, Armonk, NY, USA). Normal and approximate normal distribution of data was determined by independent sample t-test, while non-normal distribution of data was determined by Wilcoxon rank sum test. Chi-square or Fisher exact test was adopted to compare the categorical data. Receiver Operating Characteristic (ROC) curve was used to calculate the optimal cut-off value, set when the Youden index reached the maximum. The CT value means of the two groups of nodules in plain scan stage and venous stage were converted into binary data, determined by χ^2^ test. The statistically significant factors in univariate analysis were included in the binary logistic regression analysis model and the level of statistical significance was defined as *P* < 0.05.

## Results

In the clinical data, significant differences were found between patients with non-diffuse PTL and PTC in age [(63.06 ± 10.14) years old vs. (41.63 ± 15.06) years old], maximum diameter of nodule [(44.70 ± 15.50) mm vs. (31.86 ± 9.40) mm], asymmetric enlargement (74.0% vs. 57.0%) and Hashimoto's thyroiditis (78.0% vs. 23.0%), *P* < 0.05. The difference in gender ratio (male/female: 19/31 vs. 24/76) is not statistically significant (*P* = 0.074), as shown in Table 1.

**Table 1:** Comparison of clinical features between patients with non-diffuse PTL and PTC

Clinical features	PTC	PTL	Statistics	*P* value
	**(*n* = 100)**	**(*n* = 50)**		
**Sex**			χ^2^ = 3.195	0.074
Male	24 (24.0%)	19 (38%)		
Female	76 (76.0%)	31 (62%)		
**Age (years)** mean ± SD	41.63 ± 15.06	63.06 ± 10.14	t' = -10.307	< 0.001
**asymmetric enlargement**			χ^2^ = 4.118	0.042
Yes	57(57.0%)	37(74.0%)		
No	43(43.0%)	13(26.0%)		
**Hashimoto’s thyroiditis**			χ^2^ = 41.583	< 0.001
Yes	23(23.0%)	39(78.0%)		
No	77(77.0%)	11(22.0%)		
**’is the adjusted t-test value**				

**Fig. 2:**
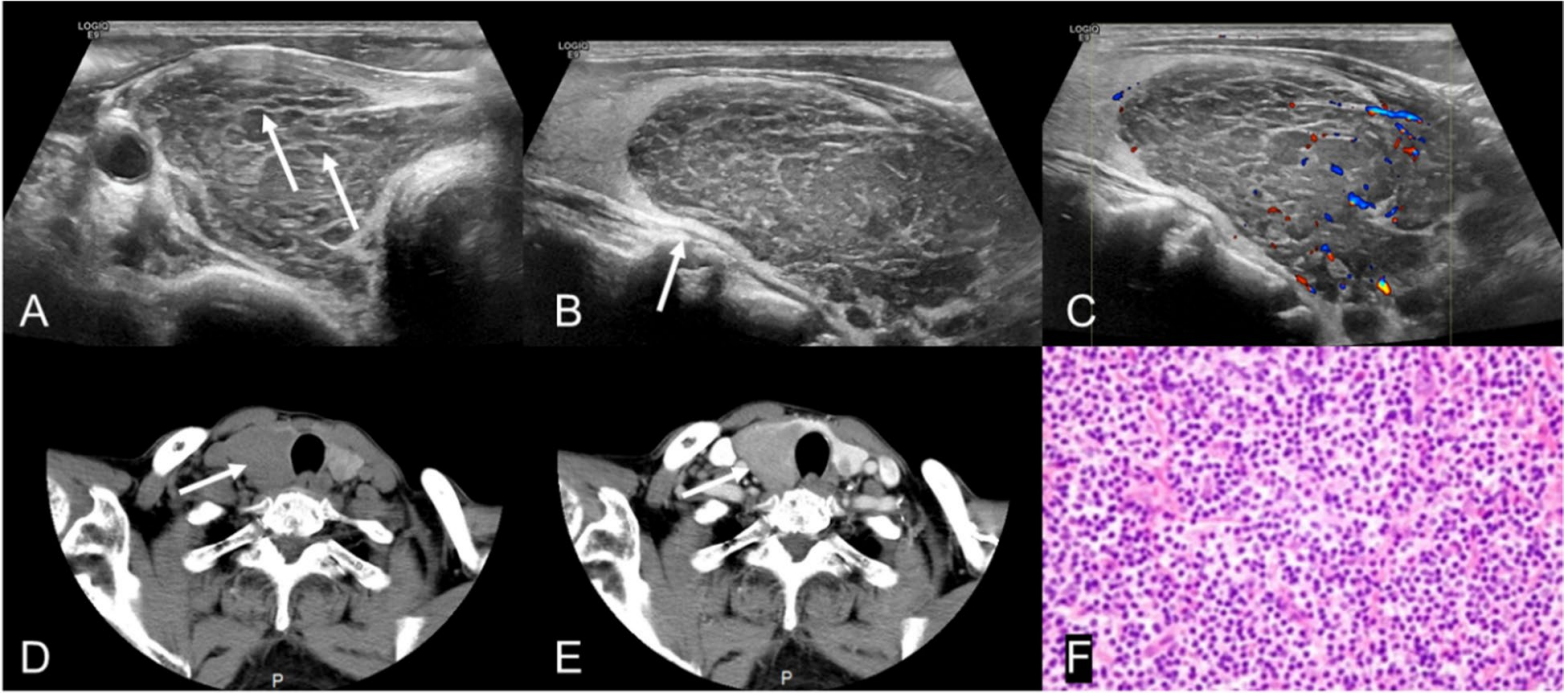
A 70-year-old female patient with neck tumor for more than 4 months (**A**) Transverse image of ultrasonography, showing a heterogeneous hypoechoic nodule with circumscribed margin on the right lobe of thyroid. The arrows show the internal linear echogenic strands; (**B**) Longitudinal image of ultrasonography. The arrow shows a significant posterior echo enhancement; (**C**) Blood flow spectrum, showing rich blood flow signals; (**D**) Non-contrast CT. The arrow shows the CT of the right lobe of thyroid; (**E**) Venous phase CT, showing homogeneous mild enhancement in the right lobe nodule and the density of it is lower than that of the thyroid parenchyma; (**F**) Pathological results, showing mucosa- associated lymphoid tissue lymphoma (MALT) (*400)

**Fig. 3:**
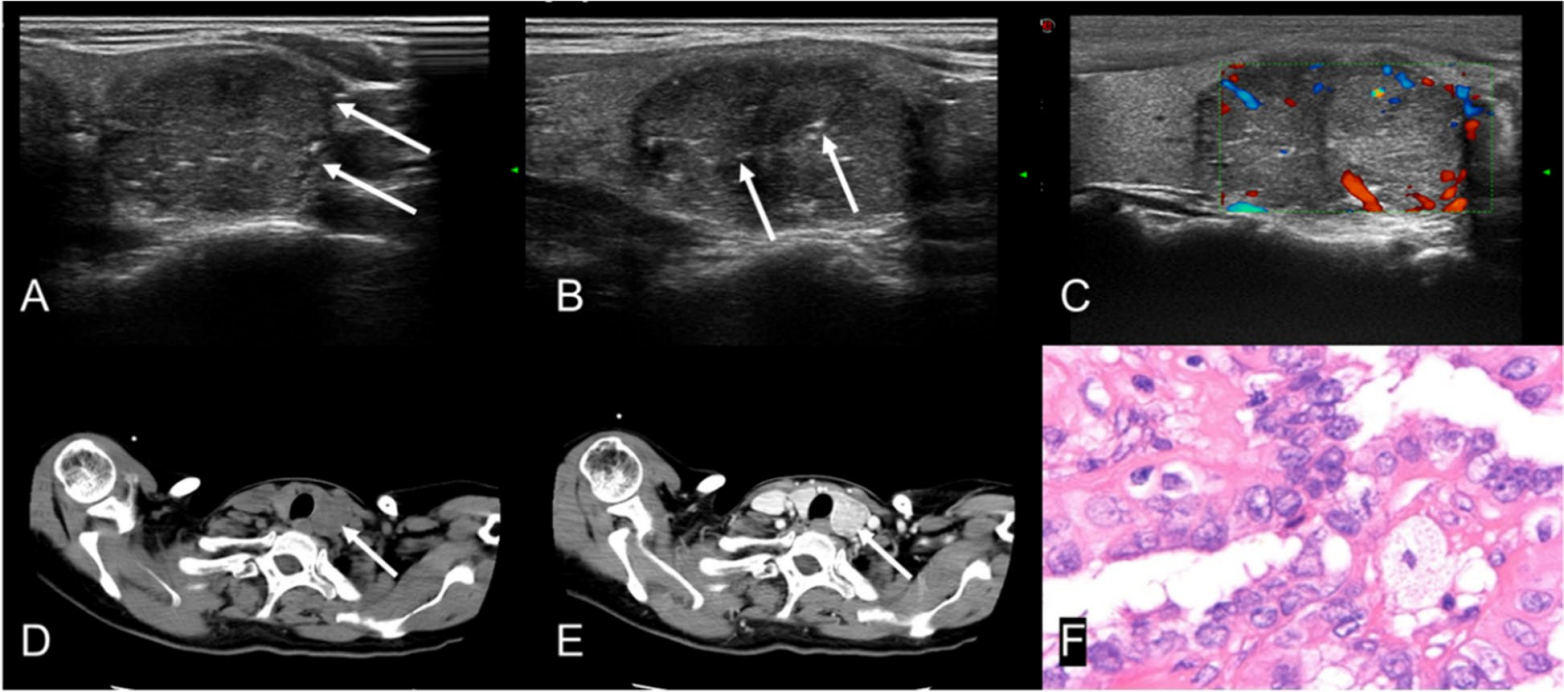
A 50-year-old female patient with neck tumor for 3 months (**A**) Transverse image of ultrasonography, showing heterogeneous hypoechoic nodules on the left lobe of thyroid with irregular border; (**B**) Longitudinal image of ultrasonography. The arrows show the internal small calcification; (**C**) Blood flow spectrum, showing rich blood flow signals; (**D**) Non-contrast CT, showing low-density nodules on the left lobe of thyroid; (**E**) Venous phase CT, showing heterogeneous significant enhancement of nodules in the left lobe of thyroid; (**F**) Pathological results, showing papillary thyroid carcinoma (* 400)

In terms of sonographic characteristics, non-diffuse PTL was higher than PTC in the markedly hypoechoic (54.0% vs. 4.3%), internal linear echogenic strands (92.0% vs. 34.0%) (Fig. 2), posterior echo enhancement (84.0% vs. 20.0%) (Fig. 2), rich vascularity (72.0% vs. 54.0%) and lack of calcification (84.0% vs. 22.0%), with a statistically significant difference (*P* < 0.05), while PTC was higher than non-diffuse PTL in irregular border (78.0% vs. 48.0%), circumscribed margin (78.0% vs. 50.0%) and capsular invasion (83.0% vs. 48.0%)(Fig. 3), with a statistically significant difference (*P* < 0.05), as shown in Table 2. Moreover, there was no significant difference between non-diffuse PTL and PTC in echo texture, cystic change and anteroposterior-to-transverse ratio (*P* > 0.05), also as shown in Table 2.

**Table 2:** Comparison of sonographic characteristics of non-diffuse PTL and PTC

Characteristic	PTC	PTL	Statistics	*P* value
	**(*n* = 100)**	**(*n* = 50)**		
**Nodule size (mm, ‾x ± s)**	31.86 ± 9.40	44.70 ± 15.50	t' = -5.384	< 0.001
**Echo texture**			χ^2^ = 0.000^*^	1.000
Homogeneous	1(1.0%)	0(0.0%)		
Heterogeneous	99(99.0%)	50(100%)		
**Echogenicity**			χ^2^ =57.429	< 0.001^a^
Markedly hypoechoic	2 (2.0%)	27 (54.0%)		
Hypoechoic	93 (93.0%)	23 (46.0%)		
Equal or high echo	5 (5.0%)	0 (0.0%)		
**Internal linear echogenic strands**			χ^2^ = 45.054	< 0.001
Yes	34(34.0%)	46(92.0%)		
No	66(66.0%)	4(8.0%)		
**Anteroposterior-to-transverse ratio > 1**			χ^2^ = 0.917^*^	0.338
Yes	10 (10.0%)	2 (4.0%)		
No	90 (90.0%)	48 (96.0%)		
**Margin**			χ^2^ = 13.787	< 0.001
Regular	22(22.0%)	26(52.0%)		
Irregular	78(78.0%)	24(48.0%)		
**Posterior echo enhancement**			χ^2^ = 56.305	< 0.001
Yes	20(20.0%)	42(84.0%)		
No	80(80.0%)	8(16.0%)		
**Cystic change**			χ^2^ = 3.043^*^	0.081
Yes	12 (12.0%)	1 (2.0%)		
No	88 (88.0%)	49 (98.0%)		
**Calcification**			χ^2^ = 52.380	< 0.001
Mirco/coarse	78 (78.0%)	8 (16.0%)		
None	22 (22.0%)	42 (84.0%)		
**Vascularity**			χ^2^ = 4.500	0.034
Absence/not rich vascularity	46 (46.0%)	14 (28.0%)		
Rich vascularity	54 (54.0%)	36 (72.0%)		
**Capsular invasion**			χ^2^ = 18.803	< 0.001
Yes	83(83.0%)	24(48.0%)		
No	17(17.0%)	26(52.0%)		

Note: a is the Fisher exact test,’ is the adjusted t-test, * is the adjusted χ2 value

In terms of CT features, non-diffuse PTL and PTC were different in the non-contrast and venous phase CT value means, with a statistical significance (*P* < 0.05) and presented in Table 3. To be exact, in these two phases the lesion density of non-diffuse PTL was lower than that of PTC. However, non-diffuse PTL was higher than PTC both in the proportion of mild to moderate enhanced intensity (80.6% vs. 27.0%) (Fig. 3) and in the proportion of homogeneous enhancement (45.5% vs. 7.0%) (Fig. 2), with a statistical significance (*P* < 0.001), also as shown in Table 3.

**Table 3:** Comparison of CT features of non-diffuse PTL and PTC

CT features	PTC	PTL	Statistics	*P* value
	**(*n* = 100)**	**(*n* = 50)**		
**non-contrast CT value (HU)mean**	47.30 ± 10.57	45.07 ± 7.19	t’ = 1.514	0.134
**Venous phase CT value(HU)mean**	111.94 ± 21.44	78.37 ± 19.94	t = 7.638	< 0.001
**non-contrast CT value**				
≤ 50.17	61 (61.0%)	25 (80.6%)	χ^2^ = 4.050	0.044
> 50.17	39(39.0%)	6 (19.4%)		
**Venous phase CT value**				
≤ 102.67	29 (29.0%)	27(90.0%)	χ^2^ = 35.019	< 0.001
> 102.67	71(71.0%)	3(10.0%)		
**Enhanced intensity**			χ^2^ = 28.449	< 0.001
Mild to moderate	27 (27.0%)	25 (80.6%)		
significant	73 (73.0%)	6 (19.4%)		
**Homogeneity**			χ^2^ = 26.578	< 0.001
Homogeneous	7(7.0%)	15(45.5%)		
Heterogeneous	93(93.0%)	18 (54.5%)		
**’ is the adjusted t-test value**				

Moreover, after the seven variables covering age, internal linear echogenic strands, posterior echo enhancement, lack of calcification, venous phase CT value mean, enhanced intensity and homogeneous enhancement were included for multivariate logistic regression analysis, this study discovered the independent predictive risk factors: age, posterior echo enhancement, lack of calcification and homogeneous enhancement, as shown in Table 4.

**Table 4:** Multivariate Logistic regression analysis of non-diffuse PTL and PTC

	**OR**	**95%CI**		***P* value**
		**Low**	**Upper**	
**Age**	1.226	1.056	1.423	**0.007**
**Internal linear echogenic strands**	1.102	0.097	12.485	0.938
**Posterior echo enhancement**	51.152	2.934	891.738	**0.007**
**Lack of calcification**	0.013	0.000	0.400	**0.013**
**Venous phase CT value**	0.084	0.003	2.145	0.134
**Enhanced intensity**	0.651	0.032	13.109	0.779
**homogeneous enhancement**	0.020	0.001	0.507	**0.018**

## Discussion

Between these two thyroid malignancies, PTC is the most common type, characterized by low malignancy, high incidence rate, good prognosis and above 90% 10-year survival rate, while despite its rarity, PTL exposes its limitations on the value of surgery and the therapeutic methods, with only two main treatments of chemotherapy and radiotherapy [28, 29]. For PTL, the 5-year survival rate of stage-IE thyroid lymphoma is as high as 75–89%, while year-year survival rate of stage-IIE thyroid lymphoma is reduced to 25–40% [1]. The prognosis of thyroid lymphoma depends on the stage [2, 30], so early diagnosis is crucial. PTL has the features of older onset age, markedly hypoechoic, internal linear echogenic strands, posterior echo enhancement, lack of calcification, rich vascularity, low CT density and homogeneous mild to moderate enhancement, while PTC retains the features of hypoechoic, irregular border, calcification, easy capsular invasion and heterogeneous significant enhancement. Ultrasound combined with CT is conducive to the diagnosis of early PTL and papillary carcinoma.

This study retrospectively reviewed the clinical and imaging features of 50 non-diffuse PTL patients and 100 PTC patients, and found that there was no significant difference in gender ratio between patients with non-diffuse PTL and PTC, both occurring commonly in the female. Comparatively, non-diffuse PTL (aged 63.06 ± 10.14 years) was greater than PTC (aged 41.63 ± 15.06 years) in the age of onset (63.06 ± 10.14 years old) with a statistical significance (*P* < 0.001), which is similar to the prior reports by Acar et al. [3] and Ota et al. [31], and was also higher than PTC in the incidence with asymmetric enlargement and HT. Studies have shown that most PTL originate in the setting of HT, with which the patients carry a 40–80 folds higher risk of thyroid lymphoma than the healthy population, and HT is the recognized risk factor for PTL [5, 32]. Of the non-diffuse PTL group (78.0%, 39/50) in this study, their pathology confirmed the evidence of HT, which is consistent with previous results reporting 30–90% PTL patients with HT [33]. However, according to the study of Mukasa [34], the incidence rate of PTC was higher than that of PTL in the patients with HT and Graves, which is inconsistent with the present result and suggested to be the influence of sample size and selection bias. Based on the significant correlation between HT and thyroid malignancy, some scholars [34, 35] proposed that the former was a precancerous lesion, which might be controversial and is expected to conduct in-depth investigation into monitoring the HT development so as to early detect, diagnose and treat PTL and PTC.

In the current study, the nodule size of non-diffuse PTL and PTC group was correspondingly 44.70 (± 15.50) mm and 31.86 (± 9.40) mm, and non-diffuse PTL lesions were obviously larger, with an average size of about 45 mm, which is similar to the reports of Gu et al. [24]. Besides, this study also found that the most common echo type of non-diffuse PTL and PTC lesions was the markedly hypoechoic and the hypoechoic respectively, and that the proportion of markedly hypoechoic was significantly higher in non-diffuse PTL than in PTC (78% vs. 4.3%). Xia et al. [11] argued that the markedly hypoechoic type in PTL as one of its typical features, might be attributed to the high consistency of its tumor cell morphology and size. The present study demonstrated that the proportion of internal linear echogenic strands was higher in non-diffuse PTL than in PTC, while previous studies [36] showed that this echo’s hypoechoic structure might result from the degree of fibrous tissue proliferation. Additionally, in our study most nodules’ posterior echo enhancement was significantly higher in non-diffuse PTL than in PTC (84%, 42/50). Logistic regression analysis indicated that this enhancement was an independent predictor of non-diffuse PTL, which is consistent with the result of Ota et al. [31], who concluded that posterior echo enhancement was a sonographic characteristic reliably distinguishing PTL from other diseases, and might be related to the large number of lymphocyte infiltration in PTL. Their subjects’ pathology revealed that lymphoma cells grew densely and homogeneously with same shape and size, which produced very small acoustic impedance difference and attenuation and finally posterior echo enhancement. Generally, posterior echo enhancement is prone to occurring in cystic nodules or benign solid tumors but rarely in the type of malignant tumors. However, once markedly hypoechoic nodule is found in the thyroid, the posterior echo will be significantly enhanced, a highly visible symptom of non-diffuse PTL.

With respect to calcification, the rate of non-diffuse PTL group in this study was only 16% (8/50), significantly lower than that of PTC group (78.0%, 78/100). Logistic regression analysis showed that lack of calcification was an independent predictor of non-diffuse PTL, which is consistent with the result of the research by Gu et al. [24] that lack of calcification was a characteristic manifestation of PTL. In contrast, no subject with calcification in PTL was found in their study, but 8 in the present research, which might be influenced by the small sample size of their study and their situation of most PTL complicated with HT.

In terms of CT features, nodules of non-diffuse PTL and PTC mostly showed a low density during the non-contrast phase, despite a lower CT value mean and a more homogeneous degree of non-diffuse PTL. However, after enhanced scanning, non-diffuse PTL tended to have a mild to moderate homogeneous enhancement, while PTC mostly showed a heterogeneous significant enhancement, with a statistically significant difference. The results show that the CT features of non-diffuse PTL in this study are consistency with those offered in previous literature [19], while these features of PTC are somewhat different from those obtained in the previous findings, such as by Kim et al. [37], who argued that the CT of PTC mostly performed with homogeneous low density, and showed a significant homogeneous enhancement after enhanced scanning, which might be related to the larger PTC nodules we chose.

Several limitations are identified in this study: (1) as a retrospective study, relatively long in the sampling period and subjective in the evaluation of sonographic and CT features; (2) small sample size and sample selection bias. Besides, the study also indicates that further improvements include collection of larger samples in the later stage, combination of new technologies in sonographic elasticity, radiography, iconography, etc.

## Conclusion

This study investigated the clinical values of sonographic and CT features in differentiating non-diffuse PTL and PTC. Both have presented their individual characteristics in sonographic and CT manifestations. The combination of the two contributes to improving the early diagnosis rate of the two diseases and providing reference for clinical diagnosis and treatment. Preoperative early diagnosis is crucial for formulating a best-laid treatment plan and improving prognosis thus to avoid unnecessary radical thyroidectomy.

## Data Availability

Our data can not be made publicly available for ethical reasons. Data are from the present study whose authors may be contacted at xudong@zjcc.org.cn or Department of Ultrasound, Zhejiang Cancer Hospital, Hangzhou, China.

## References

[1] Luo J, Huang F, Zhou P, Chen J, Sun Y, Xu F, Wu L, Huang P (2019). Is ultrasound combined with computed tomography useful for distinguishing between primary thyroid lymphoma and Hashimoto's thyroiditis?. Endokrynol Pol.

[2] Stein SA, Wartofsky L (2013). Primary thyroid lymphoma: a clinical review. J Clin Endocrinol Metab.

[3] Acar N, Acar T, Avcı A, Haciyanlı M (2019). Approach to primary thyroid lymphoma: case series. Turkish journal of surgery.

[4] Watanabe N, Noh JY, Narimatsu H, Takeuchi K, Yamaguchi T, Kameyama K, Kobayashi K, Kami M, Kubo A, Kunii Y (2011). Clinicopathological features of 171 cases of primary thyroid lymphoma: a long-term study involving 24553 patients with Hashimoto's disease. Br J Haematol.

[5] Mancuso S, Carlisi M, Napolitano M, Siragusa S: Lymphomas and thyroid: Bridging the gap. Hematological oncology 2018. 10.1002/hon.2504 29484690

[6] Ralli M, Angeletti D, Fiore M, D'Aguanno V, Lambiase A, Artico M, de Vincentiis M, Greco A (2020). Hashimoto's thyroiditis: An update on pathogenic mechanisms, diagnostic protocols, therapeutic strategies, and potential malignant transformation. Autoimmun Rev.

[7] Caturegli P, De Remigis A, Rose NR (2014). Hashimoto thyroiditis: clinical and diagnostic criteria. Autoimmun Rev.

[8] Grani G, Carbotta G, Nesca A, D'Alessandri M, Vitale M, Del Sordo M, Fumarola A (2015). A comprehensive score to diagnose Hashimoto's thyroiditis: a proposal. Endocrine.

[9] Anderson L, Middleton WD, Teefey SA, Reading CC, Langer JE, Desser T, Szabunio MM, Mandel SJ, Hildebolt CF, Cronan JJ (2010). Hashimoto thyroiditis: Part 2, sonographic analysis of benign and malignant nodules in patients with diffuse Hashimoto thyroiditis. AJR Am J Roentgenol.

[10] Liu L, Yu Y, Chen L, Zhang Y, Lu G, Gao Y (2022). Clinical differences between IgG4 Hashimoto's thyroiditis and primary thyroid lymphoma. Eur Thyroid J.

[11] Xia Y, Wang L, Jiang Y, Dai Q, Li X, Li W (2014). Sonographic appearance of primary thyroid lymphoma-preliminary experience. PLoS ONE.

[12] Lin JD, Hsueh C, Chao TC (2015). Long-Term Follow-Up of the Therapeutic Outcomes for Papillary Thyroid Carcinoma With Distant Metastasis. Medicine (Baltimore).

[13] Hay ID, Johnson TR, Thompson GB, Sebo TJ, Reinalda MS (2016). Minimal extrathyroid extension in papillary thyroid carcinoma does not result in increased rates of either cause-specific mortality or postoperative tumor recurrence. Surgery.

[14] Ywata de Carvalho A, Kohler HF, Gomes CC, Vartanian JG, Kowalski LP (2021). Predictive factors for recurrence of papillary thyroid carcinoma: analysis of 4,085 patients. Acta otorhinolaryngologica Italica : organo ufficiale della Societa italiana di otorinolaringologia e chirurgia cervico-facciale.

[15] Zheng  X, Yu S, Long J, Wei Q, Liu L, Liu C, Ren W (2022). Comparison of the clinical characteristics of primary thyroid lymphoma and diffuse sclerosing variant of papillary thyroid carcinoma. Endocrine connections.

[16] Li P, Zhang H (2019). Ultrasonography in the Diagnosis and Monitoring of Therapy for Primary Thyroid Lymphoma. Ultrasound Q.

[17] Li JW, Chang C, Chen JY, Shi ZT, Chen M (2019). Nodule Size Effect on Diagnostic Performance of Ultrasonography and Computed Tomography for Papillary Thyroid Carcinoma. Current medical imaging reviews.

[18] Raffaelli M, Tempera SE, Sessa L, Lombardi CP, De Crea C, Bellantone R (2020). Total thyroidectomy versus thyroid lobectomy in the treatment of papillary carcinoma. Gland Surg.

[19] Kim HC, Han MH, Kim KH, Jae HJ, Lee SH, Kim SS, Kim KH, Chang KH (2003). Primary thyroid lymphoma: CT findings. Eur J Radiol.

[20] Haugen BR, Alexander EK, Bible KC, Doherty GM, Mandel SJ, Nikiforov YE, Pacini F, Randolph GW, Sawka AM, Schlumberger M (2016). 2015 American Thyroid Association Management Guidelines for Adult Patients with Thyroid Nodules and Differentiated Thyroid Cancer: The American Thyroid Association Guidelines Task Force on Thyroid Nodules and Differentiated Thyroid Cancer. Thyroid : official j American Thyroid Assoc.

[21] Perez AR, Perez MEC, Arcilla CE (2020). Radical surgery for primary thyroid lymphoma in a Filipino female: Report of a case and review of literature. Int J Surg Case Rep.

[22] Ando T, Kato H (2022). Different CT imaging findings between histological subtypes in patients with primary thyroid lymphoma.

[23] Swerdlow SH, Campo E, Pileri SA, Harris NL, Stein H, Siebert R, Advani R, Ghielmini M, Salles GA (2016). The 2016 revision of the World Health Organization classification of lymphoid neoplasms. Blood.

[24] Gu LS, Cui NY, Wang Y, Che SN, Zou SM, He W, Liu JY, Gong XT (2017). Comparison of sonographic characteristics of primary thyroid lymphoma and anaplastic thyroid carcinoma. J Thorac Dis.

[25] Guo Q, Li J, Xin J, Chen X (2022). Analysis of anteroposterior-to-transverse ratio in predicting thyroid malignancy on ultrasonography. The Journal of laryngology and otology.

[26] Adler DD, Carson PL, Rubin JM, Quinn-Reid D (1990). Doppler ultrasound color flow imaging in the study of breast cancer: preliminary findings. Ultrasound Med Biol.

[27] Liu Z, Lv X, Wang W, An J, Duan F, Feng X, Chen X, Ouyang B, Li S, Singh S (2014). Imaging characteristics of primary intracranial teratoma. Acta radiologica (Stockholm, Sweden : 1987).

[28] Liu Y, Wang Y, Zhao K, Li D, Chen Z, Jiang R, Wang X, He X (2020). Lymph node metastasis in young and middle-aged papillary thyroid carcinoma patients: a SEER-based cohort study. BMC Cancer.

[29] Shirley LA, Jones NB, Phay JE (2017). The Role of Central Neck Lymph Node Dissection in the Management of Papillary Thyroid Cancer. Front Oncol.

[30] Sarinah B, Hisham AN (2010). Primary lymphoma of the thyroid: diagnostic and therapeutic considerations. Asian J Surg.

[31] Ota H, Ito Y, Matsuzuka F, Kuma S, Fukata S, Morita S, Kobayashi K, Nakamura Y, Kakudo K, Amino N (2006). Usefulness of ultrasonography for diagnosis of malignant lymphoma of the thyroid. Thyroid : official journal of the American Thyroid Association.

[32] Travaglino A, Pace M, Varricchio S, Insabato L, Giordano C, Picardi M, Pane F, Staibano S, Mascolo M (2020). Hashimoto Thyroiditis in Primary Thyroid Non-Hodgkin Lymphoma. Am J Clin Pathol.

[33] Ma B, Jia Y, Wang Q, Li X (2014). Ultrasound of primary thyroid non-Hodgkin's lymphoma. Clin Imaging.

[34] Mukasa K, Noh JY, Kunii Y, Matsumoto M, Sato S, Yasuda S, Suzuki M, Ito K, Ito K (2011). Prevalence of malignant tumors and adenomatous lesions detected by ultrasonographic screening in patients with autoimmune thyroid diseases. Thyroid : official journal of the American Thyroid Association.

[35] Bozec A, Lassalle S, Hofman V, Ilie M, Santini J, Hofman P (2010). The thyroid gland: a crossroad in inflammation-induced carcinoma? An ongoing debate with new therapeutic potential. Curr Med Chem.

[36] Nam M, Shin JH, Han BK, Ko EY, Ko ES, Hahn SY, Chung JH, Oh YL (2012). Thyroid lymphoma: correlation of radiologic and pathologic features. J Ultrasound Med.

[37] Kim DW (2014). Computed tomography features of papillary thyroid carcinomas. J Comput Assist Tomogr.

